# Evaluation of Neurodevelopmental Outcomes in Premature Twins (Multigestations) with Retinopathy of Prematurity Receiving Anti-VEGF: A Comparison Study

**DOI:** 10.1155/2022/5177401

**Published:** 2022-07-31

**Authors:** Fariba Ghassemi, Ali Makateb, Afsar Dastjani Farahani, Alireza Mahmoudi, Fatemeh Bazvand

**Affiliations:** Department of Ophthalmology, Farabi Eye Research Center, Farabi Eye Hospital, Tehran University of Medical Sciences, Tehran, Iran

## Abstract

**Purpose:**

To assess possible neurodevelopmental delay (NDD) following intravitreal antivascular endothelial growth factor (VEGF) injection in neonates with retinopathy of prematurity (ROP).

**Methods:**

In this retrospective cohort study, neurodevelopmental milestones in patients with a history of ROP and intravitreal bevacizumab (IVB) injection were compared with other gestations that received either no treatment or only a laser for treatment.

**Results:**

One hundred and twenty-five neonates (of 59 multi-gestation pregnancies) were included in this study (with the range of age 1–7 years old). Sixty-five (51.18%) were male and sixty-two (48.81%) were female. The mean gestational age (GA) and birth weight of all neonates were 29.69 ± 1.57 weeks (ranges: 26–33 weeks) and 1312.50 ± 269.33 grs (ranges: 730–2100 grs). None of the neurodevelopmental outcomes were statistically different when two subgroups in group *A* (IVB vs. control) were compared. None of the differences between IVB and laser treated subgroups is statistically significant, except for “reaching for toys,” which was delayed in the laser treated subgroup (6.6 ± 2.5 and 6.9 ± 2.5 months in IVB and laser treated subgroups, respectively).

**Conclusion:**

In neonates with ROP, there is no linear correlation between intravitreal anti-VEGF injection and neurodevelopmental delay.

## 1. Introduction

Retinopathy of prematurity (ROP) is one of the major causes of visual loss in neonates. In the late stages of the disease, neovascularization can cause traction and detachment of the underlying retina, resulting in decreased vision. The primary cause of neovascularization is an increase in vascular endothelial growth factor (VEGF) concentrations in the retina and vitreous [1, 2]. Laser retinal periphery ablation is the standard treatment for ROP type 1 as indicated in the early treatment for retinopathy in prematurity study [3].

Although laser therapy can inhibit disease progression from stage 3 to stage 4 in 90% of patients, it can destroy a large proportion of the retina. It is considered necessary to provide another therapeutic plan that will do the least amount of damage [4, 5]. The VEGF concentration in the vitreous of ROP patients is significantly increased, demonstrating the value of intravitreal anti-VEGF injection in these patients [6]. As a monoclonal humanized VEGF antibody, intravitreal injection of bevacizumab (IVB) has been proven to be an efficient treatment for ROP patients. The BEAT-ROP trial, a scientific randomized study for the use of bevacizumab in the care of patients with ROP, has shown its substantial efficacy compared to conventional laser therapy in ROP neonates with zone 1-stage 3 disease [6].

There is little information on the efficacy of IVB for neonates as at least 2800 treated patients need to be checked for a definitive inference, a large data that cannot be readily accessed [7–10]. VEGF has been shown to play an important role in neonatal neurodevelopment. The probability of systematic inhibition of VEGF and its possible detrimental effect on the neurological development of neonates treated with intravitreal anti-VEGF is unknown. Several published studies have evaluated potential side effects of anti-VEGF injection-induced neurodevelopmental delay (NDD) in human neonates and laboratory animals [11–16].

Neurodevelopment can be readily impacted by environmental and genetic influences, and studying twins and comparing them can significantly limit these possible confounding factors. This study compares this possible side effect between twin newborns, one receiving anti-VEGF and the other receiving laser therapy or no treatment.

## 2. Methods

This is a retrospective analysis of a cohort study conducted at Farabi Eye Hospital, Tehran University of Medical Science from March 2020 to August 2020. Informed consent was obtained from the parents of patients. The ethics committee of Tehran University of Medical Sciences in Farabi Eye Hospital research center approved this study and the study followed the tenets of the Helsinki Declaration.

Medical documents of all twins or multiple gestation neonates with ROP diagnosis visited at ROP clinic between 2014 and 2020 were evaluated. The patients (at the age of at least 1 at the time of the study) with IVB injection in one neonate and no treatment or only laser treatment in other gestations were included in the study. We used the multigestation patients to reduce bias in comparisons between twins.

**Figure 1:**
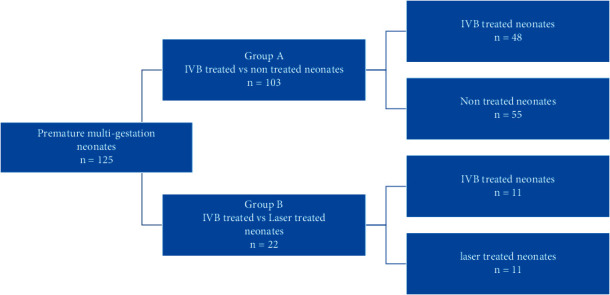
Study flowchart.

This study is on two main groups; in the first group (*A*), at least one infant has received anti-VEGF and the other one or ones have received no treatment. In the second group (*B*), one infant has received anti-VEGF and the other one or ones have received laser (Figure 1). In this retrospective cohort study, neurodevelopmental milestones in patients with a history of ROP and intravitreal bevacizumab (IVB) injection were compared with other gestations that received either no treatment (Group *A*) or only a laser for treatment (Group *B*).

Only neonates with at least stage III or plus disease who had been treated were enrolled. Stages 4 and 5 were excluded due to the fact that low vision might affect the development of patients irrespective of the type of treatment. Demographic data like birth date, gestational age, birth weight, and gestation numbers were extracted from medical documents.

Concomitant diseases and their treatments in the neonatal period like sepsis, anemia, acute respiratory distress syndrome (ARDS), intra-ventricular hemorrhage, blood transfusion, oxygen therapy with mask or ventilator, and phototherapy were also documented as possible risk factors for ROP and developmental delay.

The exclusion criteria were any ophthalmic or nonophthalmic surgery. Examples of ophthalmic disorders are congenital glaucoma or cataract, and any other chronic systemic disease such as cerebral palsy other than ROP that would affect the development of infants during the follow-up period.

A detailed comparative questionnaire based on age and stage was prepared by the supervision of a pediatrician. It was a translation of age-sex questionnaire (ASQ) as a developmental screening measure of study. In a comparative approach, a trained ophthalmology nurse was responsible for interviewing the mothers in person and helping them about the development specifics of neonates after ROP care and filling out the prepared questionnaire for each twin/triplet or quadruplet.

### 2.1. Surgical Procedure

#### 2.1.1. IVB Injection

The injection procedure was done in the operation room under topical anesthesia while the vital signs were monitored throughout the entire procedure. The eyes were prepared in a standard fashion using 5% povidone/iodine. After 5 min, a single intravitreal injection of 0.625 mg (0.025 ml) bevacizumab (Avastin, Genentech Inc., South San Francisco, CA, USA) was done through pars plicata, 1–1.5 mm posterior to the limbus in all patients. A nurse helped hold the infant during the injection. After the injection, the patients received topical antibiotics (gentamycin or sulfacetamide 10%) for 3 days. The infants were re-evaluated after 1 day and 7 days, and then every week or biweekly based on the eye's situation of patients to document the progression of the disease and any related complications. Afterward, the examinations continued until complete vascularization of the retina was observed.

#### 2.1.2. Laser

The pupils were dilated by three instillations of mydriacyl drops (tropicamide) every 15 minutes, and the neonates were pretreated with intravenous midazolam (100 *μ*g/kg) and fentanyl (3 mg/kg) by a neonatologist, who monitored them throughout the procedure. Local anaesthetic drops were added. A tight scatter of laser applications was performed, with a half burn diameter between burns, from the ridge up to the ora serrata. The whole circumference of the avascular retina was treated with moderate white burns.

### 2.2. Statistical Methods

To present data, we used mean, standard deviation, median, range, frequency, and percentage. To compare the groups regarding the possible correlation of the measurements in the same family we used a Generalized Estimating Equation (GEE). All statistical analysis was performed by SPSS (IBM Corp. Released 2017. IBM SPSS Statistics for Windows, Version 25.0. Armonk, NY: IBM Corp.). *P* value less than 0.05 is considered statistically significant.

## 3. Results

### 3.1. Demographic Data

One hundred fifty-seven medical records (from 75 multigestations pregnancies) of multigestation neonates aged at least 1 at the time of study were evaluated, one of which received anti-VEGF and the other one was treated with laser or received no treatment. Thirty patients were excluded due to association with cerebral palsy, no acceptance of parents for entering into this investigation, etc. Finally, one hundred twenty-seven neonates (from 59 multigestational pregnancies) were included in this study (aged 1 to 7).

Sixty-five (51.18%) were male and sixty-two (48.81%) were female.

The mean gestational age (GA) and birth weight of all neonates were 29.69 ± 1.57 weeks (ranges: 26–33 weeks) and 1312.50 ± 269.33 grs (ranges: 730–2100 grs), respectively.

Oxygen therapy with mask or ventilator, phototherapy, and blood transfusion were done for 80 (63%), 35 (27.6%), 73 (57.5%), and 42 (33.1%) of neonates, correspondingly. Sepsis, acute respiratory distress syndrome, and intra-ventricular hemorrhage occurred in 47 (37%), 53 (41.7%), and 3 (2.4%) of patients, respectively. The details of the zone and stage of patients are shown in Table 1.

### 3.2. IVB Treated versus Nontreated Neonates

One hundred and three multi-gestation neonates (from 48 multigestations pregnancies) were entered into this group in which at least one infant received anti-VEGF and the other one or ones received no treatment. Forty-eight were treated with IVB compared to fifty-five nontreated neonates.

Nineteen (39.6%) of IVB treated cases were male and 29 (60.4%) were female. In contrast, 33 (60%) of nontreated cases were male and 22 (40%) were female.

The mean birth GA of the group *A* was thirty weeks. Also, the mean BW of IVB treated neonates was 1316 grs in contrast to 1340 grs in the nontreated subgroup (*P*=0.722).

With the exception of ARDS, none of the concomitant medical conditions of neonates were statistically significant risk factors between these two subgroups (Table 2).

**Figure 2:**
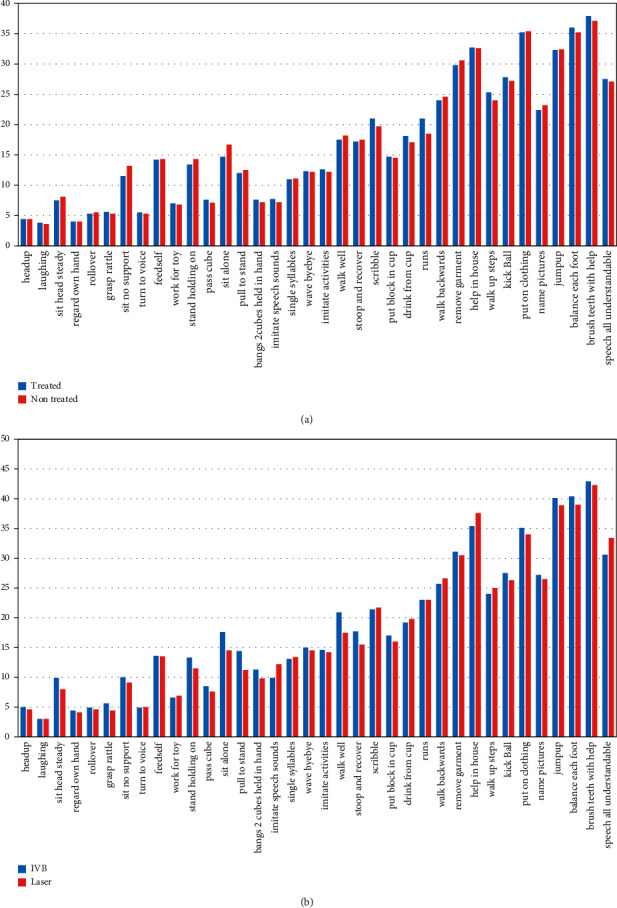
The graph of neurodevelopmental milestones of neonates in two treatment type subgroups intravitreal bevacizumab vs. no treatment in (a) and intravitreal bevacizumab vs. laser in (b).

Neurodevelopment milestones were also compared between two subgroups of group *A* (Table 3 and Figure 2(a)). In the comparison made between the two subgroups (IVB vs. control), none of the neurodevelopmental outcome differences were statistically significant.

### 3.3. IVB Treated versus Laser Treated Neonates

Twenty-two neonates (from 11 multi-gestation pregnancies) were in this group (11 neonates received IVB and 11 neonates received laser). Seven (63.6%) cases of IVB treated were male and four (36.4%) were female, in contrast to four (36.4%) of laser treated were male and seven (63.6%) were female.

The mean GA was twenty-eight weeks in this group. Also, the mean BW of IVB treated neonates was 1200 grs compared to 1208 grs in the laser-treated group (*P*=0.777).

In these two subgroups, concomitant systemic disorders in neonates were compared (Table 4) and none of them was statistically significant as a risk factor.

The neurodevelopmental milestones were also compared between two treatment subgroups (Table 5 and Figure 2(b)). In comparison between two treatment subgroups none of them were statistically significant unless “reaching for toys,” which was delayed in laser treated subgroup (6.6 ± 2.5 and 6.9 ± 2.5 months in IVB and laser treated subgroups, respectively).

## 4. Discussion

In our retrospective cohort analysis, we divided our patients into two groups: group A (IVB versus control) and group B (IVB versus laser). We didn't find any significant correlations between subgroups in the former group. In the latter, there was no substantial variation in neurodevelopmental results with the exception of “reaching for toys.”

The efficacy of intravitreal injection of anti-VEGF has been suggested by the BEAT-ROP study. Bevacizumab is an off-label treatment option in neonates with stage 3 ROP disease in many countries [4]. While its local intravitreal anti-VEGF effect is sufficient for the neonate, there are significant concerns regarding its potential detrimental effects on its neurodevelopment [7, 17]. Kong et al. evaluated the pharmacokinetics of bevacizumab and its potential effects on serum VEGF in infants with ROP. In their study, serum anti-VEGF levels were still detectable 60 days after injection, with a level peak on the 14^th^ day [15]. Furthermore, there is evidence that intravitreal anti-VEGF injection will decrease the amount of systemic plasma VEGF [1, 18]. In recent years, a number of retrospective studies have been conducted examining the potential side effect of neurodevelopmental delay in neonates after intravitreal injection of anti-VEGF, but none of them has included a comparison of neurodevelopment amongst twins [6, 8, 11, 19]. According to existing databases at the time of writing this paper, our study is the first long-term study in this era.

In this investigation, we found no significant difference between patients receiving IVB and patients without treatment for retinopathy of prematurity. The results of previous research are largely in support of our observations that no substantial association has been observed between intravitreal anti-VEGF and delay in neurodevelopment [11, 15, 16, 19].

Martinez et al. published a case series of 7 neonates evaluated by the Baley scale of infant development screening test who were treated with intravitreal bevacizumab. All 7 patients were between 11 to 28 weeks of age, 4 of which showed completely normal scores, 3 of which were presented in high-risk group for neurodevelopmental delay in different cognitive, receptive communication, fine motor and gross motor skills subgroups. At last, they have concluded that we cannot deprive neonates from the favorable effect of anti-VEGF on ROP control and we should not overlook associated diseases in these preterm infants as a confounding factor [16]. Our findings revealed no major adverse effects of IVB on the long-term neurodevelopmental consequences of premature infants compared to their twin who received no treatment. Future prospective design studies, however, are needed to validate our findings.

Two theories can be proposed for the explanation of these results. While the decreased plasma level of VEGF after intravitreal injection of IVB was well documented [1, 18], the more local VEGF-compensation level, higher exposure or more secondary impact of VEGF on organ growth may be expected. In addition, the systemic effect of IVB in premature infants may be short-term. In a study by Jo et al. in 2015, it was shown that intravitreal injection of anti-VEGF in neonate mice can transfer to systemic blood circulation and decrease brown fat density in them [12]. Despite that, they showed that the brown fat will regain its morphology and vascularity after multiple weeks by mice aging and weight gain. This poses some concerns about the possible metabolic and developmental effects of anti-VEGF on adipose tissue (brown fat) of preterm neonates with retinopathy.

Another theory that may be suggested is the greater effect of anti-VEGF on potentially harmful elevated levels of VEGF on certain neurodevelopmental characteristics of premature babies, such as the skin. Some of our results supported this hypothesis that showed insignificant earlier neurodevelopmental outcomes (“sit head steady,” “sit no support,” “stand holding on,” “sit alone,” “walk well” and “remove garment”) occurred in infants receiving IVB as compared to their own twins.

In our study, the neurodevelopmental outcomes were compared between patients receiving IVB and their twins receiving only laser treatment. There was a non-significant gap between these two subgroups. We only had 11 twins in these subgroups, and these differences might be partly explained by the low number of patients. However, only “reach for toy” appeared slightly earlier in the IVB group in comparison to the laser group. Other outcomes like “imitate speech sound” and “walk backward” occurred insignificantly earlier in IVB group than the laser group. However, some negligible results of older age existed in IVB classes, such as “sit head steady,” “sit no support,” “stand holding on,” “sit alone,” “pull to stand,” and “walk well” etc. The seriousness of eye disease (IVB injection was done in posterior zone 2 or zone 1 and more severe ROP, but laser was performed in ROP in mid to anterior zone 2 with less severity) may indicate the severity of disease in other organs such as the brain.

More research is needed to draw a definitive conclusion between these two groups. In a recent study by Raghuram et al., neurodevelopment and visual acuity were compared between two IVB and laser treated neonates with a ROP diagnosis at 18–24 months of age. Thirty-four (60 eyes) infants received IVB, and 30 (51 eyes) infants in the laser group were included. No significant differences were identified in neurodevelopment and visual function between the two groups [11].

The retrospective design and low number of patients were the two major drawbacks of our study. However, by comparing neurodevelopment scores between twins, we have dramatically minimized the potential influence of confounding factors such as gestational age and neonatal environmental factors, which may impact scores even more than the potential impact of anti-VEGF.

There was no significant association between intravitreal anti-VEGF injection and NDD in neonates with ROP, no matter the twins received anti-VEGF, laser, or no treatment.

## Figures and Tables

**Table 1:** Situation of retinopathy of prematurity of patients.

		Group *A*		Group B	
		IVB treated	Non treated	IVB treated	Laser treated
Zone	1	14	0	2	0
	2	34	27	9	11
	3	0	28	0	0

Plus	Yes	48	0	11	11
	No	0	55	0	0

Stage	Avascular area	0	4	0	0
	1	0	32	0	0
	2	3	19	1	0
	3	45	0	10	11

**Table 2:** Comparison of demographic data and systemic risk factors between IVB treated versus nontreated preamature multigestation neonates.

Parameter		Treatment		*P* value
		Yes	No	
Birth weight		1316 ± 266	1340 ± 331	0.722
Gestational age		30 ± 1 (27 to 33)	30 ± 1 (27 to 33)	1.000

Gender	Male	19 (39.6%)	33 (60.0%)	0.475
	Female	29 (60.4%)	22 (40.0%)	

Treatment	Treated	48 (100.0%)	0 (0.0%)	
	Non treated	0 (0.0%)	55 (100.0%)	

Oxygen	No	18 (37.5%)	24 (43.6%)	0.256
	Yes	30 (62.5%)	31 (56.4%)	

Ventilator	No	36 (75.0%)	39 (70.9%)	0.856
	Yes	12 (25.0%)	16 (29.1%)	

Intraventricular hemorrhage	No	47 (97.9%)	53 (96.4%)	0.653
	Yes	1 (2.1%)	2 (3.6%)	

Sepsis	No	30 (62.5%)	36 (65.5%)	0.538
	Yes	18 (37.5%)	19 (34.5%)	

Phototherapy	No	19 (39.6%)	24 (43.6%)	0.499
	Yes	29 (60.4%)	31 (56.4%)	

Transfusion	No	35 (72.9%)	37 (67.3%)	0.341
	Yes	13 (27.1%)	18 (32.7%)	

Anemia	No	44 (91.7%)	50 (90.9%)	0.453
	Yes	4 (8.3%)	5 (9.1%)	

ARDS	No	25 (52.1%)	34 (61.8%)	0.033
	Yes	23 (47.9%)	21 (38.2%)	

ARDS; acute respiratory distress syndrome.

**Table 3:** Comparison of neurodevelopmental outcomes between IVB treated versus non treated preamature multigestation neonates.

	Treated		Diff	95% CI		*P* value
	Yes	No		Lower	Upper	
Head up	4.4 ± 1.5	4.4 ± 1.3	0.039	−0.334	0.412	0.838
Laughing	3.8 ± 1.2	3.6 ± 1.3	0.069	−0.135	0.273	0.505
Sit head steady	7.5 ± 1.6	8.1 ± 3	−0.574	−1.368	0.219	0.156
Regard own hand	4 ± 1.3	4 ± 1.3	0.008	−0.197	0.214	0.937
Roll over	5.3 ± 1.3	5.5 ± 2.1	−0.321	−0.832	0.19	0.218
Grasp rattle	5.6 ± 3.2	5.3 ± 1.9	0.193	−0.475	0.861	0.572
Sit no support	11.5 ± 14.8	13.2 ± 19.1	−0.12	−1.128	0.887	0.815
Turn to voice	5.5 ± 5.5	5.3 ± 5.1	−0.013	−0.173	0.146	0.869
Feed self	14.2 ± 8.5	14.3 ± 6	−0.113	−1.418	1.192	0.865
Work for toy	7 ± 2.6	6.8 ± 2.3	0.005	−0.574	0.584	0.987
Stand holding on	13.4 ± 7.4	14.3 ± 15.1	−1.076	−5.3	3.148	0.618
Pass cube	7.6 ± 3	7.1 ± 2.5	0.23	−0.337	0.798	0.426
Sit alone	14.7 ± 3	16.7 ± 15.1	−2.004	−6.134	2.127	0.342
Pull to stand	12 ± 2.7	12.5 ± 5.4	−0.696	−1.8	0.407	0.216
Bangs 2 cubes held in hand	7.6 ± 2.5	7.2 ± 1.8	0.292	−0.246	0.831	0.287
Imitate speech sounds	7.7 ± 3.8	7.2 ± 3.3	−0.02	−0.249	0.209	0.863
Single syllables	11 ± 4.2	11.1 ± 4.3	−0.264	−1.027	0.5	0.498
Wave bye-bye	12.3 ± 3.7	12.2 ± 3.9	−0.192	−0.893	0.51	0.593
Imitate activities	12.6 ± 4.4	12.2 ± 4.5	−0.226	−0.898	0.446	0.51
Walk well	17.5 ± 3.6	18.2 ± 6.9	−0.932	−2.467	0.603	0.234
Stoop and recover	17.2 ± 3.8	17.5 ± 6.4	−0.565	−1.959	0.829	0.427
Scribble	21 ± 8.9	19.7 ± 6.2	0.753	−0.926	2.433	0.379
Put block in cup	14.7 ± 4.6	14.5 ± 4.3	0.011	−0.213	0.234	0.923
Drink from cup	18.1 ± 7.8	17.1 ± 6.1	0.424	−0.751	1.6	0.479
Runs	23.8 ± 4.5	23.6 ± 4.4	0.266	−0.56	1.091	0.528
Walk backwards	24 ± 4.8	24.6 ± 6	0.142	−0.412	0.696	0.615
Remove garment	29.8 ± 9.1	30.6 ± 8.5	−0.215	−1.264	0.833	0.687
Help in house	32.7 ± 9.3	32.6 ± 10.6	−0.155	−1.043	0.734	0.733
Walk up steps	25.3 ± 9.1	24 ± 9.6	0.143	−0.455	0.742	0.639
Kick ball	27.8 ± 8.6	27.2 ± 8.9	0.217	−0.421	0.855	0.505
Put on clothing	35.2 ± 9.1	35.4 ± 9.7	−0.345	−1.38	0.691	0.514
Name pictures	22.4 ± 7.7	23.2 ± 9.7	−0.227	−0.892	0.437	0.502
Jump up	32.3 ± 9.3	32.4 ± 9.9	−0.03	−0.582	0.522	0.916
Balance each foot	36 ± 8.2	35.2 ± 10	−0.035	−0.579	0.508	0.898
Brush teeth with help	37.9 ± 11.1	37.1 ± 11.8	−0.027	−0.079	0.025	0.312
Speech all understandable	27.5 ± 7.7	27.1 ± 7.7	0.608	−0.399	1.614	0.237

**Table 4:** Comparison of neurodevelopmental outcomes between IVB treated versus laser treated preamature multigestation neonates.

Parameter		Treated		*P* value
		Yes	No	
Birth weight		1200 ± 249	1208 ± 209	0.777
Gestational age		28 ± 1 (26 to 30)	28 ± 1 (26 to 30)	1.000

Gender	Male	7 (63.6%)	4 (36.4%)	
	Female	4 (36.4%)	7 (63.6%)	

Type of treatment	IVB	11 (100.0%)	0 (0.0%)	
	Laser	0 (0.0%)	11 (100.0%)	

Oxygen	No	2 (18.2%)	3 (27.3%)	0.300
	Yes	9 (81.8%)	8 (72.7%)	

Ventilator	No	8 (72.7%)	8 (72.7%)	1.000
	Yes	3 (27.3%)	3 (27.3%)	

Intraventricular hemorrhage	No	11 (100.0%)	11 (100.0%)	1.000
	Yes	0 (0.0%)	0 (0.0%)	

Sepsis	No	6 (54.5%)	7 (63.6%)	0.297
	Yes	5 (45.5%)	4 (36.4%)	

Phototherapy	No	4 (36.4%)	6 (54.5%)	0.126
	Yes	7 (63.6%)	5 (45.5%)	

Transfusion	No	6 (54.5%)	6 (54.5%)	1.000
	Yes	5 (45.5%)	5 (45.5%)	

Anemia	No	9 (81.8%)	9 (81.8%)	1.000
	Yes	2 (18.2%)	2 (18.2%)	

ARDS	No	7 (63.6%)	8 (72.7%)	0.298
	Yes	4 (36.4%)	3 (27.3%)	

**Table 5:** Neurodevelopment milestones between IVB treated versus laser treated preamature multigestation neonates.

	Type treatment		Diff	95% CI		*P* value
	IVB	Laser		Lower	Upper	
Head up	5 ± 2.6	4.6 ± 1.2	0.364	−0.621	1.348	0.469
Laughing	3 ± 1.1	3 ± 1.1	−1.17*E* − 17	−0.308	0.308	1.000
Sit head steady	9.9 ± 5.4	8 ± 2.1	1.909	−0.775	4.593	0.163
Regard own hand	4.4 ± 1.7	4.1 ± 1.4	0.25	−0.05	0.55	0.102
Rollover	4.9 ± 1.4	4.6 ± 1	0.273	−0.237	0.782	0.294
Grasp rattle	5.6 ± 3.9	4.4 ± 0.8	1.223	−0.692	3.138	0.211
Sit no support	10 ± 2.6	9.1 ± 2.5	0.909	−0.258	2.076	0.127
Turn to voice	4.9 ± 3.1	5 ± 3.1	0.125	−0.29	0.54	0.555
Feed self	13.6 ± 6.9	13.5 ± 6.9	0.1	−0.086	0.286	0.292
Reach for toy	6.6 ± 2.5	6.9 ± 2.5	−0.333	−0.641	−0.025	0.034
Stand holding on	13.3 ± 8.3	11.5 ± 3.7	1.727	−1.968	5.423	0.360
Pass cube	8.5 ± 4.8	7.6 ± 3.8	0.818	−0.924	2.561	0.357
Sit alone	17.6 ± 10.4	14.5 ± 2.8	3.126	−2.291	8.543	0.258
Pull to stand	14.4 ± 12.1	11.2 ± 3	3.2	−2.966	9.366	0.309
Bangs 2cubes held in hand	11.3 ± 5.6	9.8 ± 2.8	1.556	−0.891	4.002	0.213
Imitate speech sounds	9.9 ± 6.1	12.2 ± 12.8	−2.3	−11.23	6.63	0.614
Single syllables	13.1 ± 4.1	13.4 ± 7.1	−0.65	−3.672	2.372	0.673
Wave bye-bye	15 ± 6.8	14.5 ± 5.6	0.455	0.431	1.341	0.315
Imitate activities	14.6 ± 3.3	14.2 ± 3.3	0.333	−0.777	1.444	0.556
Walk well	20.9 ± 10.8	17.5 ± 4.9	3.4	−2.94	9.74	0.293
Stoop and recover	17.7 ± 8.7	15.5 ± 3.6	2.273	−2.367	6.913	0.337
Scribble	21.4 ± 8	21.7 ± 8.3	−0.222	−0.494	0.049	0.109
Put block in cup	17 ± 8.1	16 ± 6.5	1	−3.928	5.928	0.691
Drink from cup	19.2 ± 7.1	19.8 ± 9.5	−0.636	−4.485	3.212	0.746
Runs	23 ± 7.9	23 ± 6.7	−0.333	−1.022	0.355	0.343
Walk backwards	25.7 ± 1.5	26.6 ± 4.1	−0.285	5.514	4.944	0.915
Remove garment	31.1 ± 10.2	30.5 ± 11.4	0.545	−4.343	5.434	0.827
Help in house	35.4 ± 14	37.6 ± 14.7	−0.857	−2.413	0.698	0.280
Walk up steps	24 ± 6.4	25 ± 6.9	−1.099	−3.423	1.225	0.354
Kick ball	27.5 ± 12	26.3 ± 10.1	1.2	−4.006	6.406	0.651
Put on clothing	35.1 ± 12.8	34 ± 12.1	1.091	−2.556	4.738	0.558
Name pictures	27.2 ± 16.9	26.5 ± 15.5	0.727	−4.3	5.755	0.777
Jump up	40.1 ± 12	38.9 ± 12.9	−0.857	−2.412	0.698	0.280
Balance each foot	40.4 ± 17	39 ± 16.3	0	0	0	0.350
Brush teeth with help	42.9 ± 13.4	42.3 ± 14.1	6.39*E* − 05	−9.21*E* − 05	0	0.422
Speech all understandable	30.6 ± 14.5	33.4 ± 14.4	−2.5	−6.585	1.585	0.230

## Data Availability

Data will be available if requested on reasonable cause.
